# A Case of Vanishing Bronchus Syndrome in a Non-lung Transplant Patient

**DOI:** 10.7759/cureus.50168

**Published:** 2023-12-08

**Authors:** Diane S Habib, Ariana Azimi-Shooshtari, Siva T Sarva, Ramesh Kesavan, Gnananandh Jayaraman

**Affiliations:** 1 Internal Medicine, Hospital Corporation of America (HCA) Houston Healthcare-Kingwood/ University of Houston College of Medicine, Kingwood, USA; 2 Pulmonary Critical Care, Pulmonary Critical Care and Sleep Specialist, Kingwood, USA; 3 Pulmonary and Critical Care Medicine, Hospital Corporation of America (HCA) Houston Healthcare-Kingwood/ University of Houston College of Medicine, Kingwood, USA

**Keywords:** non-hodgkin’s lymphomas, non-lung transplant, radiation pneumonitis, endobronchial ultrasound (ebus), vanishing bronchus syndrome

## Abstract

Vanishing bronchus syndrome (VBS) is the most severe form of bronchial stenosis. It has been described as a complication following a lung transplant (LT). We present a case of VBS in a patient with non-Hodgkin lymphoma in remission status post chemotherapy and radiation therapy and no history of a lung transplant.

## Introduction

Airway complications (AC) following a lung transplant (LT) include but are not limited to, bronchial stenosis, dehiscence, infections, and tracheobronchomalacia [1]. Bronchial stenosis is associated with increased morbidity and mortality. It is the most common AC, reported in up to 32% of cases [2]. Affected patients can be asymptomatic or present with post-obstructive pneumonia and an obstructive pattern on pulmonary function testing [1]. Vanishing bronchus syndrome (VBS) has been described as the most severe form of bronchial stenosis following LT, causing a complete obliteration of the bronchial lumen. It accounts for nearly 2% of those cases and occurs at variable times, usually months later, likely secondary to ischemic and/or infectious processes distal to the anastomoses [3, 4]. We describe a case of VBS in a patient with no history of LT.

## Case presentation

This is a case report of a 54-year-old Caucasian female who is an active smoker (13 pack-years) with a medical history of chronic obstructive pulmonary disease (COPD), cardiovascular disease, stage III chronic kidney disease, autoimmune hepatitis, and non-Hodgkin lymphoma status post chemotherapy and radiation therapy 15 years ago. She is currently in remission and was initially admitted for necrotizing pneumonia.

**Figure 1:**
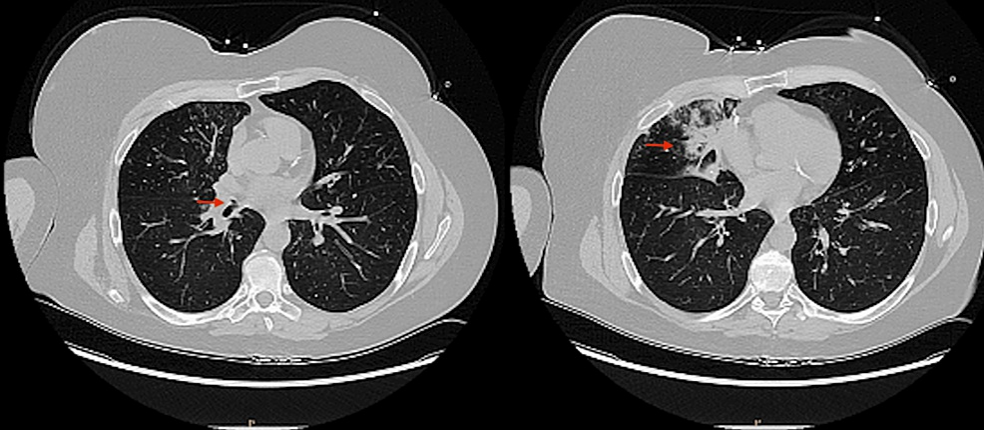
Chest CT images showing occlusion at the right middle lobe bronchus origin with consolidation/cavitation (red arrows)

The patient presented with four days of right pleuritic chest pain and a dry cough associated with fever, nausea, and vomiting. A chest CT without contrast (Figure 1) showed an occlusion of the right middle lobe (RML) bronchus at the origin, with consolidation or cavitation in the RML.

**Figure 2:**
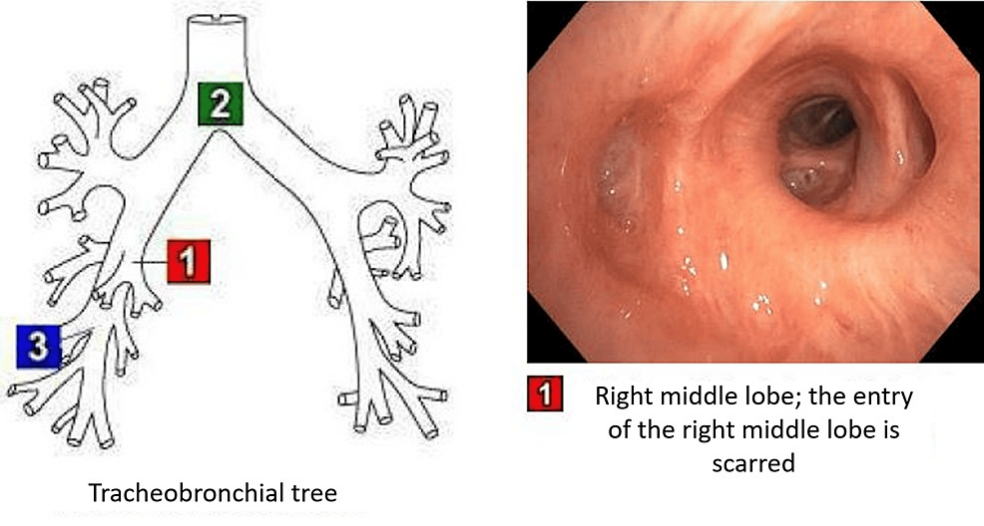
View of the stenosed right middle lobe opening from a flexible bronchoscopy

Sputum cultures, a viral respiratory panel, *Legionella pneumophila* urinary antigen test, and *Streptococcus pneumoniae* antigen test were negative. Further investigation with a flexible bronchoscopy was warranted. The RML opening appeared completely closed and cicatrized (Figure 2).

Bronchoalveolar lavage cultures were negative. Endobronchial ultrasound bronchoscopy (EBUS) revealed similar findings of RML entry occlusion and no evidence of extrinsic compression. An elongated lymph node in station seven was biopsied and was negative for malignancy.

Pathology revealed no evidence of malignancy or viral/fungal changes. Interventional pulmonology was consulted, and an attempt to open the RML opening was not successful. The patient completed a course of antibiotics with the resolution of the consolidation on repeat imaging.

## Discussion

Vanishing bronchus syndrome is the most severe form of bronchial stenosis that typically occurs after LT [4]. There is no clear cause for VBS. However, some etiologies include an ischemic injury or an immune-mediated process [5]. Radiation-induced lung injury (RILI) has been described in the literature and is a dose-limiting toxicity. Radiation pneumonitis is the early phase of RILI and occurs secondary to inflammation and desquamation of the alveolar epithelium and endothelium. Symptoms during this phase can vary in intensity, be non-specific, and usually happen in the first three to 12 weeks following radiation [6, 7]. Radiation pneumonitis eventually evolves, causing radiation fibrosis [8]. Fibrosis starts six to eight months after exposure to radiation with fibroblast activation, and the process continues over the years [6, 7, 9]. This effect is further potentiated by some chemotherapy drugs with known direct pulmonary toxicity or radiation-enhancing effects (including cyclophosphamide, doxorubicin, and vincristine) [10]. Although our patient did not undergo LT, she had radiation therapy and was exposed to cyclophosphamide and vincristine as part of her treatment. Though radiation fibrosis occurs mainly at the alveoli level, this patient developed RML scarring 15 years after exposure. This is likely the result of an extensive inflammatory, ischemic, and immune process that was triggered by the chemotherapy and radiation therapy and progressed, resulting in VBS.

## Conclusions

Vanishing bronchus syndrome has been recorded as a known airway complication in the transplanted lung and not in other clinical settings. This case sheds light on the potential for patients with no history of LT to develop VBS. In particular, clinicians should maintain a high degree of suspicion for VBS in cases without a history of LT, especially in the setting of RILI. Early pulmonary intervention may be warranted in the form of balloon dilatation and stenting to salvage the lobe and prevent post-obstructive pneumonia. Further investigations with retrospective and prospective studies are warranted.
